# Identification and analysis of unitary loss of long-established protein-coding genes in Poaceae shows evidences for biased gene loss and putatively functional transcription of relics

**DOI:** 10.1186/s12862-015-0345-x

**Published:** 2015-04-18

**Authors:** Yi Zhao, Liang Tang, Zhe Li, Jinpu Jin, Jingchu Luo, Ge Gao

**Affiliations:** State Key Laboratory of Protein and Plant Gene Research, College of Life Science, Center for Bioinformatics, Peking University, Beijing, 100871 People’s Republic of China; Current address: College of Horticulture and Landscape Architecture, Southwest University, Chongqing, 400715 People’s Republic of China; State Key Laboratory of Systematic and Evolutionary Botany, Institute of Botany, Chinese Academy of Sciences, Beijing, 100093 People’s Republic of China

**Keywords:** Unitary gene loss, Poaceae, Competitive endogenous RNA

## Abstract

**Background:**

Long-established protein-coding genes may lose their coding potential during evolution (“unitary gene loss”). Members of the Poaceae family are a major food source and represent an ideal model clade for plant evolution research. However, the global pattern of unitary gene loss in Poaceae genomes as well as the evolutionary fate of lost genes are still less-investigated and remain largely elusive.

**Results:**

Using a locally developed pipeline, we identified 129 unitary gene loss events for long-established protein-coding genes from four representative species of Poaceae, i.e. brachypodium, rice, sorghum and maize. Functional annotation suggested that the lost genes in all or most of Poaceae species are enriched for genes involved in development and response to endogenous stimulus. We also found that 44 mutated genomic loci of lost genes, which we referred as relics, were still actively transcribed, and of which 84% (37 of 44) showed significantly differential expression across different tissues. More interestingly, we found that there were totally five expressed relics may function as competitive endogenous RNA in brachypodium, rice and sorghum genome.

**Conclusions:**

Based on comparative genomics and transcriptome data, we firstly compiled a comprehensive catalogue of unitary gene loss events in Poaceae species and characterized a statistically significant functional preference for these lost genes as well showed the potential of relics functioning as competitive endogenous RNAs in Poaceae genomes.

**Electronic supplementary material:**

The online version of this article (doi:10.1186/s12862-015-0345-x) contains supplementary material, which is available to authorized users.

## Background

Both point mutations and large deletions can disrupt open reading frame of long-established protein-coding genes in a species or a clade, resulting in the loss of coding potential and genetic functions, which is referred to as “unitary gene loss” [1-4]. Although it is taken as “deleterious” by conventional wisdom, several studies demonstrated that unitary gene loss may also contribute to evolutionary novelty and even be adaptive, e.g. immune responses [1] and pathogen interactions [5,6] in humans, the origin of a partially reproductively isolated race in *Drosophila melanogaster* [7,8], and the generation of self-fertilisation in *Arabidopsis* [9]. Several systematic profiles for unitary gene loss have been done in human and rodents [1-4]. Interestingly, Marques et al. found more than half of lost genes in rodents retained expression and played a new role as competitive endogenous RNAs (ceRNAs) to regulate the expression of other transcripts by altering microRNA availability [4], which suggests a possible functional mechanism for these relics.

Members of the Poaceae are a major food source, and this family represents an ideal model clade for plant evolutionary analysis [10]. Recently, two genome-wide studies investigated loss of duplicated gene copies in plants [11,12]. However, the global picture of unitary loss for long-established protein-coding genes in Poaceae remain largely unknown and elusive.

In this study, we first developed a novel comparative genomics-oriented pipeline to identify unitary loss of long-established gene in the four representative Poaceae, i.e. brachypodium, rice, sorghum and maize. Taking *Arabidopsis*, poplar and grape as out groups, we focused on lineage-specific loss of long-established protein-coding genes which have been conserved for more than 160 million years ago (Mya) [13] and only got lost after divergence of Poaceae clade. Employing stringent criteria, we identified 129 unitary gene loss events (UGLEs) in the four genomes, with 47, 27, 23 and 32 UGLEs identified in brachypodium, rice, sorghum and maize genome, respectively. Expression profiling analysis showed that the relics of 44 lost genes were still actively transcribed, 37 of which showed significantly varied expression pattern across different tissues. After removing these more likely due to partially degenerated promoters, we still identified 30 reliable expressed relics. Among them, we identified one brachypodium relic, two rice relics and two sorghum relics as putative ceRNAs, suggesting that unitary gene loss may contribute to the origin of functional non-coding RNAs.

## Results

### Identification of unitary gene loss events

Unitary loss of long-established protein-coding genes can be identified by both orthologous mapping of protein sequences [1,3], and syntenic mapping of gene loci [2,4]. To date, most studies take pseudogenes, i.e. relics, as proxies for identifying gene loss events [14]. However, if the gene loss occurred long ago or evolved very rapidly, it would be difficult to identify relics based on homologous searching alone. Moreover, if a gene is lost through intra-chromosomal recombination [15], it is also hard to detect relics based on left homologous fragments. In addition, syntenic mapping cannot identify lost genes without significant synteny, inducing false-negative cases caused by genome rearrangement such as insertion, deletion and translocation event [16].

**Figure 1:**
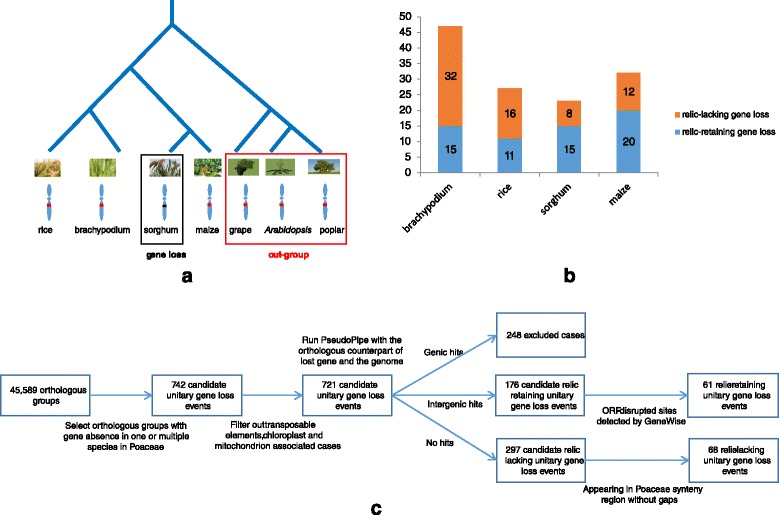
Identification of unitary gene loss in Poaceae species. **(a)** An example of unitary gene loss identification in sorghum. Orthologous genes are absent in sorghum but present in all other Poaceae species and all three out-group species; therefore, a candidate unitary gene loss event has been called in sorghum. In addition, orthologous genes in other species are regarded as orthologous counterparts of the lost gene. **(b)** Number of unitary gene loss events, including relic-retaining and relic-lacking ones, in four species of Poaceae, i.e., brachypodium, rice, sorghum and maize. **(c)** The entire pipeline for unitary gene loss identification. The number of candidates retained at each filtering step is shown.

To address these challenges, we developed a novel pipeline independent of relics and synteny to identify unitary gene loss events in Poaceae. Using this pipeline, we firstly identified candidate unitary gene loss events based on orthologous relationships among different species. Three out groups including *Arabidopsis*, poplar and grape were used and the principle of parsimony [17,18] was adopted to distinguish gene loss events from gene gains in other lineages (Figure 1a, also see Methods for more details).

Beginning with 45,589 orthologous groups containing orthologous genes in four Poaceae species and three out-group species, we identified 742 candidate unitary gene loss events. In the case of possible false-positives caused by transposable elements or mitochondrial and chloroplast-related genes, we further removed 21 cases, in which 7, 2, 4 and 8 from brachypodium, rice, sorghum and maize respectively, and generated a dataset containing 721 candidate unitary gene loss events (see Methods for more details).

To further filter out false-positive unitary gene loss events due to incorrectly annotated orthologous relationships, we ran PseudoPipe [19] using the entire genome sequence of the species as the object and protein sequence of an orthologous counterpart of the lost gene from the adjacent species (e.g., brachypodium for rice) as the query. We then extracted genomic hits for 424 candidate unitary gene loss events. Of these events, 248 candidates with genic hits were removed, because they may primarily occur due to incomplete genomic annotation rather than *bona fide* unitary gene loss. For the rest 176 candidates that matched intergenic regions, we removed an additional 115 events that had intact open reading frame (ORF) matches. The remaining 61 disabler-harbouring candidates, which contains at least one ORF-disrupting site, such as a frameshift or premature stop codon, were classified as relic-retaining unitary gene loss events. Furthermore, the remaining 297 candidate unitary gene loss events without genomic hits between the genome and orthologous counterparts are taken to analysis for validation. Only candidates which can be located in the genome based on synteny mapping across brachypodium, rice, sorghum and maize are considered as relic-lacking unitary gene loss events. After excluding candidates in synteny-inconsistent and gapped regions, we obtained 68 relic-lacking unitary gene loss events.

Finally, we identified 129 unitary gene loss events, including 61 relic-retaining and 68 relic-lacking ones (Figure 1b). Most unitary gene loss events occurred recently and only in one species, except for the unitary gene loss in orthologous group 11453 occurred before the divergence of brachypodium and rice and unitary gene loss in orthologous group 8968 occurred before the divergence of sorghum and maize. The entire pipeline is in Figure 1c while available at the website http://gene-loss.cbi.pku.edu.cn, and information on the identified unitary gene loss events is included in Additional file 1, providing a resource for the gene loss community in plants.

### Functions of lost genes in wild and cultivated species are different

The biological functions of lost genes can be inferred by their orthologous counterparts [3]. Based on ortholog mapping, we functionally annotated 124 out of 129 lost genes using Plant GO slim terms of their *Arabidopsis* orthologs retrieved from AmiGO (see Additional file 2). Subsequently, we took GO slim distribution among genes in each Poaceae species as the background and conducted GO slim enrichment analysis through Fisher’s Exact test and FDR multiple test correction, to see whether there are any functional preferences of lost genes (see Methods for more details, Table 1).

**Table 1:** **Plant GO slim enrichment of lost genes in Poaceae species**

**Class**	**Plant GO slim annotation**	**Species**	**Observed lost number**	**Expected lost number**	**Observed/Expected**	**FDR-corrected pvalue**
**Shared by four species**	multicellular organismal development	brachypodium	13	2.9	4.6	0.0000
		rice	6	0.9	6.7	0.0009
		sorghum	5	1.1	4.4	0.0133
		maize	6	1.4	4.4	0.0070
	cellular component organization	brachypodium	13	2.9	4.5	0.0000
		rice	6	1.8	3.4	0.0135
		sorghum	5	1.1	4.7	0.0126
		maize	11	1.7	6.3	0.0000
**Shared by three species**	post-embryonic development	brachypodium	9	1.6	5.5	0.0001
		sorghum	4	0.7	6.1	0.0126
		maize	6	0.8	7.7	0.0006
	response to endogenous stimulus	brachypodium	7	1.7	4.2	0.0037
		rice	4	0.6	7.1	0.0049
		maize	5	0.9	5.8	0.0060
**Shared by two species**	anatomical structure morphogenesis	brachypodium	12	1.1	11.2	0.0000
		rice	4	0.3	12.9	0.0009
	cell cycle	brachypodium	5	0.4	11.3	0.0003
		sorghum	4	0.2	21.5	0.0004
**Species-specific**	biosynthetic process	brachypodium	15	8.3	1.8	0.0300
	cell differentiation	brachypodium	8	0.6	12.4	0.0000
	cell growth	brachypodium	4	0.6	6.7	0.0076
	embryo development	brachypodium	5	0.6	8.6	0.0010
	flower development	brachypodium	8	0.6	13.6	0.0000
	growth	brachypodium	7	0.7	9.6	0.0000
	response to external stimulus	brachypodium	7	1.7	4.0	0.0045
	sequence-specific DNA binding transcription factor activity	brachypodium	7	1.9	3.6	0.0076
	carbohydrate metabolic process	rice	5	0.9	5.8	0.0035
	transport	maize	8	3.3	2.4	0.0412

First of all, lost functions in all or most of species are commonly enriched with development, such as multicellular organismal development (GO:0007275, FDR-corrected p-value = 0.000017 in brachypodium, FDR-corrected p-value = 0.00095 in rice, FDR-corrected p-value = 0.013 in sorghum and FDR-corrected p-value = 0.0070 in maize) and post-embryonic development (GO:0009791, FDR-corrected p-value = 0.00013 in brachypodium, FDR-corrected p-value = 0.012 in sorghum and FDR-corrected p-value = 0.00065 in maize). Detailed inspection showed that most of lost genes annotated as multicellular organismal development and post-embryonic development were associated with reproductive process, such as vegetative to reproductive phase transition of meristem (GO:0010228), embryo development ending in seed dormancy (GO:0009793), seed dormancy process (GO:0010162) and seed germination (GO:0009845). In addition to development, lost functions in most of species are also commonly enriched with response to endogenous stimulus (GO:0009719, FDR-corrected p-value = 0.0037 in brachypodium, FDR-corrected p-value = 0.0049 in rice and FDR-corrected p-value = 0.0060 in maize). Further inspection showed most of this kind of lost genes involved in response to varieties of hormones (i.e. auxin, gibberellin, cytokinin, abscisic acid, ethylene, jasmonic acid and brassinosteroid) and chitin.

On the other hand, we also found several species-specific functional enrichment for lost genes. The function of sequence-specific DNA binding transcription factor activity (GO:0003700, FDR-corrected p-value = 0.0076) is enriched in lost genes of brachypodium uniquely. By checking the annotation of *Arabidopsis thaliana* orthologs of this kind of lost genes in plant Transcription Factor Database (PlantTFDB) [20], we found that lost genes encoded transcription factors which are important for development of plant, such as ones in WOX and MYB families. Another species-specific enriched function of lost genes in brachypodium is response to external stimulus (GO:0009605, FDR-corrected p-value = 0.0045). Most of this kind of genes involved in response to environmental stress and phytopathogen, such as anthocyanin accumulation in tissues in response to UV light (GO:0043481), cellular process regulating host cell cycle in response to virus(GO:0060154), defense response to bacterium (GO:0042742) and response to other organism (GO:0051707). Interestingly, genes involved in the carbohydrate metabolic process (GO:0005975) prefer to be lost only in rice genome (FDR-corrected p-value = 0.0035), while transport genes (GO:0006810) are enriched in lost genes of maize (FDR-corrected p-value = 0.0412).

### Several relics are still under active transcription

Although relics remaining after unitary gene loss have lost their protein-coding potential and thus cannot function as proteins, studies demonstrated that they may still being transcribed and play other roles in the species at the transcript level, such as non-coding RNAs [21]. To explore this phenomenon in Poaceae, we detected expression signatures for 61 identified relics.

**Figure 2:**
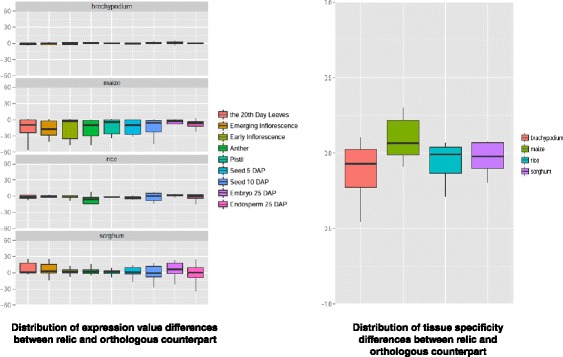
Distribution of differences associated with expression value and tissue specificity between relics and orthologous counterparts in Poaceae species.

We used four RNA-seq datasets with the same samples in four Poaceae species, including leaves, emerging inflorescence (pre-pollination tassel and pre-emergence cob for maize), early inflorescence (post-pollination tassel and post-emergence cob for maize), anther, pistil (mature silk and ovule for maize), seeds harvested 5 days after pollination (DAP), seeds harvested 10 DAP, embryo harvested 25 DAP and endosperm harvested 25 DAP to identify expressed relics [22,23]. We found there were 12 out of 15 relics in brachypodium, 9 out of 11 relics in rice, 13 out of 15 relics in sorghum and 10 out of 20 relics in maize expressed respectively. Compared with their orthologous counterparts, these expressed relics possess similar expression level and tissue specificity (Figure 2; see Additional file 3). Interestingly, we also found 11 out of 12 expressed relics in brachypodium, 9 out of 9 expressed relics in rice, 12 out of 13 expressed relics in sorghum and 5 out of 10 expressed relics in maize expressed with significant variation among different samples.

**Figure 3:**
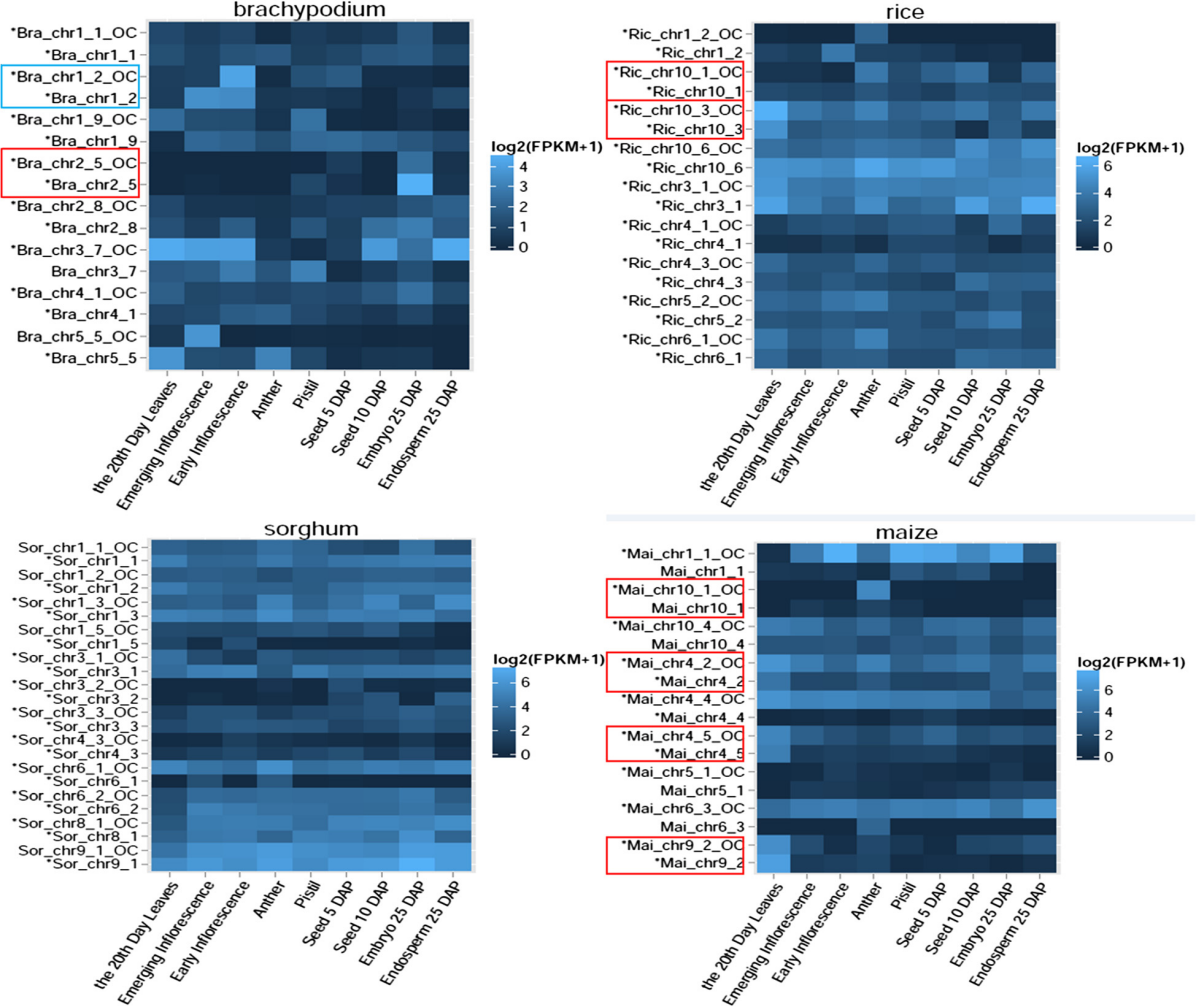
Expression signatures detected for relics and orthologous counterparts in Poaceae species. The log_2_ (FPKM + 1) values of expressed relics and orthologous counterparts in four species of Poaceae are shown in these heat maps. The orthologous counterpart in the adjacent species is selected for this expression analysis. For different species, each panel represents each dataset. The SRA accession number of RNA-seq dataset is SRP008505 for brachypodium, SRP008821 for rice, SRP008469 for sorghum and SRP006463 for maize. The vertical axis represents the number of the orthologous group where unitary gene loss occurred (OC means orthologous counterpart of the relic), and the horizontal axis represents the different samples in each dataset. The label “*” is shown if the relic or its orthologous counterpart shows significant differential expression between at least one pair of samples (FDR-adjusted pvalue < 0.05, Cuffdiff). The expression correlation of the relic and its orthologous counterpart is significantly high if they are enclosed in the red frame (Pearson’s R Correlation test, p-value < 0.05), while the conservation of the relic promoter is high if it is enclosed in the blue frame (two-tailed Wilcoxon Rank Sum test, FDR-adjusted p-value < 0.01). Only when both of the relic and its orthologous counterpart have expression signatures, their expression values are shown in this figure.

The transcription of recently evolved relics may be spurious due to their partially degenerated promoter regions [24,25]. To test this possibility, we first directly compared the expression pattern between the relic and its orthologous counterpart in the neighbouring species. After excluding four brachypodium relics, one sorghum relic and one maize relic, whose orthologous counterparts have no expression signatures detected in these four data sets, we found the expression of only seven relics (one for brachypodium, two for rice and four for maize) showed significant similar pattern with that of their orthologous counterpart (two-tailed Student’s *t* test, Pearson R > 0.6, FDR-corrected p-value < 0.05) (Figure 3; see Additional file 4). Besides comparison the expression pattern between relic and its orthologous counterpart, we also checked the conservation of promoter region of expressed relics. We identified there was one brachypodium relic possessing significantly conserved promoter region than protein-coding genes through Wilcoxon Rank Sum test (GERP score, FDR-corrected p-value < 0.05) (Figure 3; see Additional file 4). These eight relics with similar expression pattern or conserved promoters were removed from further analysis.

It has been reported that transcripts losing coding potential could function as ceRNAs in rodents [4,26]. We found there were one brachypodium relic, two rice relics and two sorghum relics encode different microRNA response elements and could be a plausible target for corresponding microRNAs, implying that they might become ceRNAs and thereby function as microRNA decoys [27,28]. Consistent with the hypothesis [4,26,29], we found there were protein-coding genes harbouring the same microRNA response elements in three prime untranslated region and expressing with positive correlation for each of these relics (Table 2). Further check showed that there were no out-paralog relationships between these relics and protein-coding genes, thus the sharing of the same microRNA response element is not due to the homology between them, further suggesting the possible ceRNA roles for these transcribed relics.

**Table 2:** **List of relics as potential ceRNAs**

**ID**	**miRNA**	**Orthologous group of relic**	**Gene with the same microRNA response element**	**Expression correlation**	**FDR-corrected pvalue for the correlation**
Bra_chr4_1	bdi-miR5181a-3p	7462	BRADI2G55497	0.89	0.00
Bra_chr4_1	bdi-miR5181a-3p	7462	BRADI3G27520	0.85	0.01
Bra_chr4_1	bdi-miR5203	7462	BRADI1G77247	0.59	0.04
Bra_chr4_1	bdi-miR5203	7462	BRADI2G55497	0.89	0.00
Ric_chr5_2	osa-miR530-5p	578	LOC_Os01g16180	0.83	0.02
Ric_chr1_2	osa-miR531b	395	LOC_Os08g35210	0.77	0.02
Sor_chr1_5	sbi-miR5384	6806	Sb01g018950	0.76	0.04
Sor_chr1_1	sbi-miR5568f-5p	6026	Sb08g015530	0.78	0.02

## Discussion

Based on orthologous relationships in Poaceae with out-group species in monocotyledons and the application of filtering criteria, we identified a total of 129 UGLEs of long-established protein-coding gene in four Poaceae genomes, including 61 relic-retaining ones and 68 relic-lacking ones. The inclusion of out-group species in monocotyledons can ensure the long-established status of lost genes and make it possible to identify unitary gene loss based on the principle of parsimony.

**Figure 4:**
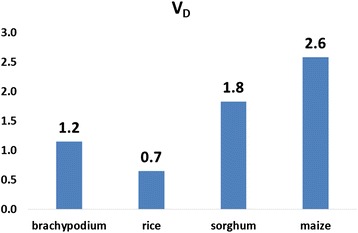
Gene death rate in Poaceae species. The unit is events/million years. *V*_*D*_, the gene death rate.

To minimize false positive rate, we adopted very strict criteria for detecting unitary gene loss in this study. For relic-retaining unitary gene loss, we filtered out those cases with evident genic matches or that lacked ORF-disrupting sites. For relic-lacking unitary gene loss, we filtered out those cases that could not be located based on synteny data. Of these unitary gene loss events, we found that 46, 26, 22 and 31 events specifically occurred in brachypodium, rice, sorghum and maize, respectively. Considering that the divergence time of brachypodium-rice is 40 million years [30] and that of sorghum-maize is 12 million years [31], we estimated the gene death rate (*V*_*D*_) for each species after the latest divergence. We found that the average death rate was 1.6 genes per million years, with the lowest (0.7) rates observed in rice and the highest (2.6) rates occurring in maize (Figure 4). Consistent with previous observations [11], the *V*_*D*_ for brachypodium (1.2) was more than 70% higher than that of rice, which may contribute to the relatively small genome size of brachypodium [30] and suggest on-going gene deletion happened in brachypodium [11]. Moreover, except for brachypodium, the accelerated gene death rate is accompanied by large genome size, suggesting the potential mechanism that large genome is less stable than small one and possesses faster evolved gene content in Poaceae [32,33].

Based on Plant GO slim enrichment analysis, both common and species-specific functional preferences of lost genes were detected in Poaceae. Being consistent with previous observation in human genome [3], the functions of development associated with the reproductive process are enriched in all of these four Poaceae genomes. Interestingly, we noticed that Schnable et al. have speculated that a special case of gene loss (the reciprocal loss of homeologous genes) may contribute to the radiation of Poaceae lineage but failed to find supporting evidence [11], while Muir and Hahn have suggested the reciprocal gene loss contributed less to increased speciation rates [34]. Our observation on the enrichment of reproductive function in unitarily lost genes, on the other hand, may provide a new angle for the hypothesis.

Besides the function of associated with reproductive process, the function associated with response to endogenous stimulus is also enriched in brachypodium, rice and maize. Commonly, most of lost genes annotated with this function are related with response to varieties of plant hormones, the key factor for plants to regulate signalling pathways involved in response to varieties of biotic and abiotic stresses [35]. Interestingly, we also found genes involved in response to endogenous chitin stimulus, a proxy for fungal infection [36,37], lost in brachypodium and maize genome, suggesting a potential role of gene loss in the adaption of changing environments stresses like biotic stresses.

There are also multiple species-specific functional preference of lost genes in different Poaceae species, especially in brachypodium. In brachypodium, genes with the molecular function as sequence-specific DNA binding transcription factor activity are preferred to lose. Detailed analysis showed that lost genes in this class are annotated as transcription factors essential for plant development based on their orthologous counterpart in *Arabidopsis thaliana* (see Additional file 5). It suggests that brachypodium reshapes its mode of development associated with reproductive process via adjustment the gene content associated with transcriptional regulation. Moreover, genes associated with response to external stimulus are also preferred to lose in brachypodium. It is consistent with the fact that brachypodium is a wild plant, which faces more rapid changing of environmental stress and phytopathogen. Therefore, similar with genes processing the function of response to endogenous stimulus, this kind of genes in brachypodium are also becoming the evolutionary hot spots.

It has been demonstrated that some relics retained after gene loss could continue to express and function as non-coding RNAs [21,38-42]. However, “functional expression” of relics as non-coding RNA must be distinguished from “leaky expression” of relics generated from partially degenerated promoter region. In *Arabidopsis* and rice, it has been demonstrated that recently lost duplicated genes could retain significantly lower expression [24,25]. However, in our data set, we found expressed relics possess similar expression level and expression width compared with their orthologous counterparts. Moreover, we also found 37 out of 44 expressed relics possess significantly varied expression values across different samples, implying their expression is under control of specific regulation. The similar expression level, expression width of relics compared with their orthologous counterparts and the specific regulation of expression suggest their expression is not likely just transcriptional noise [43].

On the other hand, the differential expression can also solely result from the left over regulatory sequence (“partially degenerated promoter”). To test the hypothesis, we further compared the expression pattern between relic and its orthologous counterpart across different samples, and assessed the evolutionary conservation of expressed relics’ promoters directly. We found the expression of more than 65% (30 out of 44) relics cannot be fully explained by the partially degeneration promoter hypothesis, implying neofunctionalization of these relics [44,45]. Interestingly, within these 30 reliable expressed relics, we found there were one brachypodium relic, two rice relics and two sorghum relics may function as ceRNAs and undertake the role of microRNA decoys, suggesting a source of novel non-coding RNAs.

## Conclusions

Based on orthologous relationships and several strict filtering criteria, we systematically identified the unitary loss of long-established protein-coding genes in four representative species of Poaceae, i.e. brachypodium, rice, sorghum and maize, discovering 129 unitary gene loss events in total. By Plant GO slim enrichment analysis, we found genes with function of development associated with reproductive process, while response to endogenous stimulus are commonly preferred to lose in all or most of Poaceae species, suggesting unitary gene loss might affect the features of reproduction and stimulus response in different Poaceae species. In addition, after excluding expressed relics are more likely due to partially degenerated promoter region, we totally assured 30 expressed relics which may bring evolutionary novelty to the species. Furthermore, among these 30 expressed relics, we found one brachypodium relic, two rice relics and two sorghum relics may function as ceRNAs, indicating a potential contribution of unitary gene loss to the origin of functional non-coding RNAs. Systematic identification and analysis of the unitary loss of long-established protein-coding genes in these four species will elucidate the global perspective and unique features of this evolutionary process in Poaceae.

## Methods

### Identification of unitary gene loss

Preliminary unitary loss of long-established protein-coding gene was identified based on the orthologous relationships among four representative species in Poaceae, i.e., brachypodium, rice, sorghum and maize, and three species in out-group, i.e., *Arabidopsis*, poplar and grape. The orthologous groups are retrieved from Ensembl Plants release 15 [46] including genes annotated in *Brachypodium distachyon* v1.0, *Oryza sativa Japonica* MSU6, *Sorghum bicolor* v1.0, *Zea mays* AGPv2, *Vitis vinifera* IGGP_12x, *Arabidopsis thaliana* TAIR10 and *Populus trichocarpa* JGI2.0.

The absence of orthologous genes in a species may either represent gene loss in that species or gene gain in other species. However, based on the principle of parsimony, if orthologous genes are present in multiple species but absent in just one, a single gene loss event is more plausible than multiple simultaneous gene gain events [17,18]. This strategy needs more species to be considered. Therefore, we assigned preliminary unitary gene loss events based on the orthologous relationships between Poaceae species brachypodium, rice, sorghum and maize, and out-group species *Arabidopsis*, poplar and grape*.* Briefly, if a gene is absent in one Poaceae species but its orthologous present in all other Poaceae and out-group species, a preliminary UGLE will be assigned to the branch, and all functional genes in the same orthologous group will be taken as the orthologous counterparts of this lost gene. In case of false positive, preliminary UGLE was further filtered by removing relics with intact ORF identified by GeneWise [47], relics with genic hits of an orthologous counterpart from the adjacent species, as well as transposable, mitochondrial and chloroplast elements.

If there were no any genomic hits of orthologous counterparts on the genome for a preliminary lost gene, its genomic location was inferred based on the synteny among Poaceae species produced by Schnable et al. [11]. In case of artefacts due to incomplete genome sequence/annotation, we excluded all preliminary relic-lacking UGLE with either gene annotation or genomic gaps in the synteny regions of neighbour genomes from further analysis.

### Plant GO slim annotation and analysis of the functional preferences of lost genes

Because there were no direct functional annotations for the lost gene, the original function of lost gene was annotated by Plant GO slim of its orthologous counterparts in *Arabidopsis.* Then, GO slims of protein-coding genes in each Poaceae species were extracted as the background for statistics test. Through Fisher’s Exact test and FDR multiple test correction, enriched GO slims were identified for lost genes in each Poaceae species.

### Detection of expression signatures for relics

Four RNA-seq datasets (i.e. SRP008505 for brachypodium, SRP008821 for rice, SRP008469 for sorghum and SRP006463 for maize) were retrieved from NCBI were used to detect expression signatures for relics. All RNA-seq reads of each dataset were mapped to the genome using TopHat [48] and called differential expression by Cuffdiff [49]. Only relic with fragments per kilobase of sequence per million reads mapped (FPKM) value greater than one in at least one sample was considered as expressed relic. Wilcoxon Signed Rank test and FDR correction were conducted to determine whether there were significant differences of the expression level and tissue specificity (i.e. the maximum value of fractional expression of locus in one tissue relative to the sum of all tissues) between relic and its orthologous counterpart.

### Conservation analysis of the promoter region for relics

The 2 kb intergenic region upstream of the 5’ boundary of relic or protein-coding gene was taken as the putative promoter. GERP (Genomic Evolutionary Rate Profiling) conservation scores [50,51] of the promoter region of relic were calculated based on multiple alignment of promoter region of relic and its orthologous counterparts, which is available at the website http://gene-loss.cbi.pku.edu.cn, and a species tree constructed via RAxML [52,53]. The tree was used as the input of GERP program to calculate the neutral rate by summing all of the lengths of the tree. To evaluate whether the conservation of the promoter region of relic was significantly different from that of protein-coding gene, GERP scores of the promoter region of protein-coding genes in the same species were also calculated and considered as the background of statistics test.

### Analysis of ceRNA features for relics

The microRNA response element (MRE) on relic and 3’ untranslated region (3’ UTR) of protein-coding gene was predicted by TAPIR [54] with the default parameters. The microRNA sequences were retrieved from miRBase release 20 [55], while the 3’ UTR sequences of protein-coding genes were retrieved from BioMart of Ensembl Plants release 15 [56]. Then, Pearson’s R Correlation test and FDR multiple test correction were conducted to identify expressed relic possessing significantly positive expression correlation with protein-coding gene harbouring the same MRE on 3’ UTR.

### Availability of supporting data

The multiple alignment of promoter region of relic and its orthologous counterparts is available in the website http://gene-loss.cbi.pku.edu.cn/, while all other supporting data is available as additional files.

## Additional files

Additional file 1:
**Detail List of unitary gene loss events in Poaceae clade.**
Additional file 2:
**Plant GO slim and GO annotation for lost genes.**
Additional file 3:
**Wilcoxon Signed Rank test result for expression value and tissue specificity between relic and its orthologous counterpart.**
Additional file 4:
**Expression correlation between the relic and its orthologous counterpart, as well as the GERP score of relic promoter.**
Additional file 5:
**Lost transcription factors in brachypodium.**

## Abbreviations

ceRNA: Competitive endogenous RNA
Mya: Million years ago
UGLE: Unitary gene loss event
ORF: Open reading frame
DAP: Days after pollination
V_*D*_: Gene death rate
FPKM: Fragments per kilobase of sequence per million reads mapped
GERP: Genomic Evolutionary Rate Profiling
MRE: microRNA response element
3’ UTR: 3’ untranslated region
FDR: False discovery rate
